# Increasing Transfection of Human Monocyte-Derived Dendritic Cells by Optimizing Lipid Nanoparticle Ionizable Lipid and mRNA Uridine Modification

**DOI:** 10.3390/pharmaceutics18040403

**Published:** 2026-03-25

**Authors:** Izabella Lambart, Daniel Flender, Dana Liu, Jenny Hong Hoang, Max Hesse, Diana Campillo-Davo, Eva Lion, Jonas Van Audenaerde, Stefan Schiller, Johanna Simon, Simon Geissler, Evelien Smits, Karsten Mäder, Hannah Zaryouh

**Affiliations:** 1 Global Drug Product Development, Merck Healthcare KGaA, 64293 Darmstadt, Germany; izabella.lambart@merckgroup.com (I.L.);; 2 Faculty I of Natural Sciences, Institute of Pharmacy, Martin Luther University Halle-Wittenberg, 06108 Halle (Saale), Germany; karsten.maeder@pharmazie.uni-halle.de; 3 Laboratory of Experimental Hematology (LEH), Vaccine & Infectious Disease Institute (VAXINFECTIO), Faculty of Medicine and Health Sciences, University of Antwerp, 2610 Antwerp, Belgium; 4 Life Science Services, Merck Life Science KGaA, 64293 Darmstadt, Germany; 5 Center for Oncological Research (CORE), Integrated Personalized and Precision Oncology Network (IPPON), Faculty of Medicine and Health Sciences, University of Antwerp, 2610 Antwerp, Belgium

**Keywords:** lipid nanoparticles, ionizable lipid, eGFP mRNA, uridine modification, human dendritic cell, transfection efficiency, electroporation

## Abstract

**Background/Objectives**: Lipid nanoparticles (LNPs) are a prominent example of delivery systems that are used to prevent the degradation of messenger ribonucleic acid (mRNA) and facilitate cell uptake. Improving LNP transfection efficiency requires careful selection of key formulation components, including the ionizable lipid and the coding sequence of the nucleic acid. Therefore, it is crucial to assess various options for the target cells, as results can differ significantly between cell types. Building on previous work investigating the effect of apolipoprotein E4 on LNP transfection of human monocyte-derived dendritic cells, we assess the impact of different ionizable lipids and compare modifications in the mRNA uridine to further optimize the delivery to these cells. **Methods**: LNPs containing eGFP mRNA with different uridine modifications were produced via microfluidic mixing and investigated for their in vitro transfection efficiency of human monocyte-derived dendritic cells. Transfection occurred in the presence of apolipoprotein E4 for different encapsulated mRNA concentrations. Delta mean fluorescence intensity and eGFP positive cells were measured by flow cytometry 48 h after transfection. Cell viability was assessed via AnnexinV/7-AAD staining, after comparing this method to LIVE/DEAD^TM^ Fixable Near-IR staining. **Results**: This study shows that a combination of SM-102 as the ionizable lipid with eGFP mRNA containing N1-methylpseudouridine enabled the transfection of human monocyte-derived dendritic cells with very high efficiency at low concentrations, allowed for dose sparing, and even led to the LNPs outperforming a specifically tailored electroporation protocol. **Conclusions**: Improvement of nucleic acid delivery to human monocyte-derived dendritic cells, known for their difficulty to be transfected, was achieved by LNP formulation tuning.

## 1. Introduction

Therapeutic approaches with messenger ribonucleic acid (mRNA) have been investigated for different diseases over the past years. Compared to DNA-based therapies, they show less potential for toxicity and gene insertion, since mRNA functionality does not depend on entering cell nuclei [1]. Nevertheless, as a negatively charged macromolecule, mRNA cannot easily pass through the anionic cell membrane and arrive in the cytoplasm to be translated into proteins. Different strategies to facilitate mRNA delivery to cells of interest have been investigated and developed to overcome challenges related to their negative charge and susceptibility for degradation, either by their inherently unstable nature or by ribonucleases [2,3].

Amongst common methods used for mRNA delivery are electroporation (EP) or lipid nanoparticles (LNPs) [4]. EP has been explored for small interfering RNA (siRNA) and mRNA and allows for a direct delivery to the cytoplasm of immune cells, increasing antigen expression and immunogenicity [5]. The potential of EP for vaccination purposes has been extensively investigated for many decades, showing EP to successfully deliver mRNA to dendritic cells (DC) and leading to some pre-clinical and clinical studies [6,7,8,9,10,11,12]. Furthermore, a study comparing different transfection methods for mRNA delivery to DC reported EP as superior to mRNA lipofection or passive mRNA pulsing [13]. In 2020, EP was still considered the most traditional physical method for human primary DC transfection, showing good transfection efficiency while maintaining high cell viability [14]. Therefore, EP can be considered a relevant standard for primary human DC transfection and serve as a positive control in studies investigating different means to deliver nucleic acids to these immune cells.

Compared to EP, LNPs have a lower cytotoxic potential and can be used in vivo because, unlike EP, they do not require an electric field or high voltage currents to momentarily disrupt the cell membrane and allow for delivery [15,16]. LNPs are typically composed of an ionizable lipid, an amphiphilic phospholipid, cholesterol, and polyethylene glycol lipids, and can be manufactured using several production methods, including microfluidic mixing [17,18]. The lipid composition can impact biodistribution and therapeutic effects, requiring careful optimization and selection for therapeutic success [19].

Besides the ionizable lipid, another key component in the formulation is the nucleic acid sequence, which directly impacts transfection efficiency success [20,21,22]. Substitution of nucleic acid bases with chemically modified versions has been investigated for many years and led to important discoveries related to mRNA stability and the reduction of innate immune responses to transfected mRNA [23]. Incorporating modifications helps achieve higher stabilization of the mRNA transcript, leading to higher protein expression, as shown for some COVID-19 mRNA vaccines substituting every non-modified uridine nucleoside with N1-methylpseudouridine (N1MePsU) [24]. This highlights the importance of investigating and comparing different uridine modifications to increase transfection efficiency, especially when the delivery targets cells known to be hard to transfect, such as immune cells.

Nucleic acid delivery to DC is known to be difficult when using traditional transfection methods [22]. Considering that enhanced DC transfection efficiency in vitro can reduce the in vivo dose of LNP-mRNA vaccines [25], we believe it is important to investigate way of improving nucleic acid delivery to these cells. LNP-mediated delivery to murine DC has already been investigated in a few studies, as reported by recent reviews [26,27]. A study reported an increase in the transfection efficiency after LNP-mediated delivery of eGFP mRNA with N1MePsU when compared to the non-modified eGFP mRNA. Conversely, substituting the non-modified uridine (U) with 5-methoxyuridine (5moU) led to a decrease in transfection efficiency [22]. As for the ionizable lipid, a study comparing MC3, ALC-0315, and SM-102 showed how SM-102 performed better in terms of mRNA transfection and protein expression efficiencies in an in vitro setup using murine primary bone marrow-derived DC or the DC2.4 mouse DC line [28]. Although murine and human DC share many similarities [29], we believe that studies with human DC are important to confirm findings and should be further pursued. A recent study using different influenza virus mRNA either unmodified or with the N1MePsU modification compared formulations containing OF-02, cKK-E10, or SM-102 as the ionizable lipid for the transfection of human peripheral blood-derived immature DC. In this study, OF-02 performed better than cKK-E10 and SM-102 during in vitro transfection assays at doses of 5 or 15 µg mRNA/10^6^ cells. Interestingly, no significant differences between mRNA sequence uridines were observed for percentage of Sing15 HA positive cells treated with the SM-102 formulation, and the highest value of around 70% was achieved only at a high dose of 15 µg/10^6^ cells [30]. Another study targeting human monocyte-derived immature DC (hiDC) concluded that apolipoprotein E4 (ApoE) facilitated LNP-mediated in vitro delivery but also used high doses of mRNA in LNPs (2 and 8 µg/10^6^ cells) [31]. Interestingly, these results were contrary to the findings of a study on murine DC, which showed no influence of ApoE on transfection [22].

This work aimed to increase the transfection efficiency of hiDC and lead to lower necessary doses of LNP-delivered mRNA in vitro as an initial step into reducing future in vivo doses of LNP-mRNA vaccines. We focused on combining LNP formulation optimization of the ionizable lipid with eGFP mRNA uridine modification. The three ionizable lipid benchmarks from the approved LNP formulations of Onpattro^®^ (MC3) and the two COVID-19 vaccines (ALC-0315 and SM-102) were selected and compared to define the best candidate. Two different uridine modifications of the eGFP mRNA sequence were compared to the unmodified uridine to identify the best combination between the investigated LNP components. The final optimized formulation was used for a dose sparing investigation with low doses of LNP-delivered eGFP mRNA (0.1 to 1 µg/10^6^ cells) and compared against the gold standard EP.

## 2. Materials and Methods

### 2.1. Materials

(6Z,9Z,28Z,31Z)-Heptatriaconta-6,9,28,31-tetraen-19-yl 4-(dimethylamino) butanoate (D-Lin-MC3-DMA) was bought from AmBeed, Arlington, VA, USA. 2-hexyl-decanoic acid, 1,1′-[[(4-hydroxybutyl)imino]di-6,1-hexanediyl] ester (ALC-0315) and 8-[(2-hydroxyethyl)[6-oxo-6-(undecyloxy)hexyl]amino]-octanoic acid, 1-octylnonyl ester (SM-102) were bought from Cayman Chemical, Ann Arbor, MI, USA. 1,2-distearoyl-sn-glycero-3-phosphocholine (DSPC) was bought from Avanti Polar Lipids Inc., Alabaster, AL, USA. 3β-Hydroxy-5-cholesten, 5-Cholesten-3β-ol (cholesterol, Sigma Grade ≥ 99%), 1,2-Dimyristoyl-rac-glycero-3-methoxypolyethylene glycol-2000 (DMG-mPEG-2000), Triton^TM^ X-100, citric acid monohydrate, nuclease-free water (Calbiochem OmniPur^®^ water, Burlington, MA, USA), ethanol and human serum albumin were bought from Merck KGaA, Darmstadt, Germany. RNase- and DNase-free sucrose was bought from VWR Life Science, part of Avantor Inc., Radnor, PA, USA. CleanCap^®^ EGFP mRNA (89% integrity by Fragment Analyzer and 99% capping efficiency by LCMS, according to manufacturer), CleanCap^®^ EGFP mRNA (5moU, 90% integrity by Fragment Analyzer and 99% capping efficiency by LCMS, according to manufacturer) and CleanCap^®^ M6 EGFP mRNA (N1MePsU, 88% integrity by Fragment Analyzer and 99% capping efficiency by LCMS, according to the manufacturer) were bought from TriLink Biotechnologies, San Diego, CA, USA. UltraPure^TM^ Tris-HCI buffer (1 mol/L and pH 7.5), human apolipoprotein E4 recombinant protein, PeproTech^®^, phosphate-buffered saline (PBS) buffer, L-glutamine, sodium pyruvate and penicillin/streptomycin, and cell medium RPMI 1640 were bought from Thermo Fisher Scientific, Waltham, MA, USA. PGE2 and TNFα were bought from Bio-Techne, Düsseldorf, Germany.

### 2.2. LNP Formulation and Production

LNPs were produced by mixing the ionizable lipid, DSPC, cholesterol and DMG-mPEG-2000 (50:10:38.5:1.5) in ethanol with nucleic acid in citrate buffer (50 mM, pH 4) using the Sunshine (previously known as Automated Nanoparticle System, Unchained Labs, Royston, UK), with a total flow rate (TFR) of 9 mL/min, a flow rate ratio (aqueous:ethanolic) of 3:1, a total lipid concentration of 20 mM, N/P ratio of 6 and the junction chip 190 µm (Unchained Labs, Royston, UK). LNP production buffer was exchanged using the Amicon^®^ Ultra centrifugal filters (Merck KGaA, Darmstadt, Germany). LNPs were diluted 15-fold inside the centrifugal filter using 20 mmol/L of Tris HCl buffer and centrifuged at 4 °C and 1000× *g* for one hour. The steps of diluting 15-fold and centrifuging for 1 h were repeated two more times. LNPs were diluted to the desired concentration of encapsulated nucleic acid with 20 mmol/L of Tris HCl buffer containing sucrose calculated for a final sucrose mass fraction of approximately 0.1. LNPs were stored frozen at −80 °C, except for fresh LNP samples used for mRNA integrity, which were stored at 4 °C until analysis.

### 2.3. LNP Characterization

#### 2.3.1. Particle Size and Distribution Analyses

Hydrodynamic diameter and polydispersity index (PDI) were measured by dynamic light scattering (Zetasizer Nano ZS, Malvern Panalytical GmbH, Kassel, Germany). Samples were diluted to approximately 1 µg/mL mRNA in LNPs using a in 20 mmol/L Tris-HCl buffer and measured backscatter mode (173°), 3 × 10 × 10 s at 25.0 °C using cumulants fit (z-average and PDI). ζ-potential was measured by laser Doppler anemometry (same instrument). LNP samples were diluted to approximately 10 µg/mL mRNA in LNPs using a 10 mM NaCl solution (resulting pH of 7.3) and measured three times at 25.0 °C in a folded capillary cell (DTS1070, Malvern Panalytical, Malvern, UK) with automatic measurement duration and voltage selection, using the Smoluchowski model and General Purpose mode. Dispersion pH was measured in a 780 pH Meter equipment attached to a LL Biotrode 3mm WOC (Metrohm, Herisau, Switzerland).

#### 2.3.2. Endotoxin Concentration

Endotoxin concentration was measured using the Endosafe Nexgen-PTS (Charles Rivers Laboratories, Wilmington, MA, USA) and calculated as EU per µg mRNA. Samples were diluted 100-fold in endotoxin free water (Charles Rivers Laboratories) and analyzed in Endosafe^®^ LAL Cartridges with 0.5–0.005 EU/mL sensitivity (product code PTS55005F, Charles Rivers Laboratiories).

#### 2.3.3. Nucleic Acid Analyses

Encapsulation efficiency (EE) and total nucleic acid recovery (TnaR) were obtained by fluorescence analysis with the Quant-iT^TM^ RiboGreen^TM^ RNA Assay Kit (Invitrogen, Thermo Fisher Scientific, Carlsbad, CA, USA). Briefly, LNPs were incubated in 96-well plates (FluoroNunc^TM^ F96 MicroWell^TM^ plate, Thermo Fisher Scientific) at 37 °C for 10 min in TE buffer, either pure or containing 1% Triton^TM^ X-100, to determine free nucleic acid (fNA) and total nucleic acid (tNA) concentrations, respectively. For the calculation of TnaR, theoretical nucleic acid concentration was used (theorNA), which describes the calculated concentration expected at the end of LNP production, assuming 100% recovery. All pipetting steps were performed by the pipetting robot Dragonfly discovery (SPT Labtech, Cambridgeshire, UK).

EE (%) = (tNA − fNA)/tNA × 100(1)

TnaR (%) = tNA/theorNA × 100(2)

Integrity of mRNAs was assessed using a Fragment Analyzer 5300 and the Standard Sensitivity RNA Kit (Agilent, Santa Clara, CA, USA). LNP samples were diluted for approximately 20 µg/mL mRNA in a solution of 20% Triton^TM^ X-100 and 30% ethanol in water. The mixture was incubated for 20 min at 30 °C and 600 rpm (Thermomixer C, Eppendorf, Hamburg, Germany) before mixing of 2 µL with 22 µL RNA Diluent Marker solution (15 nt) in 96-well PCR plates. Plates were mixed for 2 min followed by an incubation for 2 min at 70 °C (Mastercycler, Eppendorf). Directly after heating, plates were cooled down to 4 °C and mixed with a plate spinner (VWR PCR Plate Spinner, VWR, Radnor, PA, USA) prior to measurement. The method DNF-471-33–SS Total RNA was used to assess the integrity of mRNA, either unformulated or in LNP samples.

### 2.4. In Vitro Assays

hiDC were manufactured as described previously [32]. Briefly, buffy coats from healthy volunteer donors were obtained via the Red Cross Flanders [10], which is responsible for the collection of human blood and obtains informed consent from all donors, including consent for the use of their material for scientific research. From these buffy coats, peripheral blood mononuclear cells were isolated, after which CD14+ monocytes were purified and subsequently differentiated into hiDC. All use of human blood-derived material in this study was approved by the Ethics Committee of the University of Antwerp (Antwerp, Belgium) and the Antwerp University Hospital (Antwerp, Belgium) under the reference number 5488. ApoE (Thermo Fisher Scientific, Cranbury, NJ, USA) was dissolved in PBS buffer to a concentration of 50 µg/mL and stored at −20 °C until further use. hiDC were seeded into a 6-well plate (1 × 10^6^ cells/well) and incubated at 37 °C with eGFP mRNA LNPs in 1 mL RPMI 1640 medium (Thermo Fisher Scientific, Paisley, UK) in the presence of the maturation cytokines PGE_2_ (1 µg/mL, Tocris Bioscience, Bristol, UK)) and TNFα (20 ng/mL, Miltenyi Biotec, Bergisch Gladbach, Germany) without serum for 2 h. Afterwards, an equal volume of RPMI 1640 containing 5% human serum albumin (Merck Millipore, Darmstadt, Germany), 2% penicillin/streptomycin (Thermo Fisher Scientific, Grand Island, NY, USA), 2% L-glutamine (Thermo Fisher Scientific, Paisley, UK), 2% sodium-pyruvate (Thermo Fisher Scientific, Paisley, UK), 1 µg/mL PGE_2_, and 20 ng/mL TNFα was added to the plate to bring the final serum concentration to 2.5%. LNPs were thawed at room temperature before addition to the wells to achieve the desired concentration of eGFP mRNA encapsulated in LNPs. ApoE was added to achieve 1 µg/well immediately before transfection with LNPs. EP using free 2 µg eGFP mRNA was performed on a Gene Pulser Xcell device (Biorad, Hercules, CA, USA) for 2 × 10^6^ cells/well, as previously described [10]. For cell viability studies, hiDC were either harvested with PBS-EDTA, washed with FACS buffer (BD FACSFlowTM Sheath Fluid + 0.1% BSA (*w*/*v*) + 0.05% NaN_3_ (*w*/*v*) and stained for 15 min at room temperature with LIVE/DEAD^TM^ Fixable Near-IR (Thermo Fisher Scientific, Eugene, OR, USA), or harvested with PBS-EDTA, washed with PBS and stained with 7-AAD (Biolegend, San Diego, CA, USA) and Annexin V-APC (BD Biosciences, San Jose, CA, USA) in 1× Annexin V Binding Buffer (BD Biosciences) for 15 min at 4 °C. This staining was used to distinguish viable cells from those undergoing early/late apoptosis. The Annexin V^−^/7-AAD^−^ population was considered as the percentage of viable hiDC (gating strategies are described in Figures S1 and S2). Acquisition was performed on a NovoCyte Quanteon (Agilent Technologies). Fluorescence signal was measured by flow cytometry (Novocyte Quanteon) 48 h post-transfection and analyzed with FlowJoTM v10.10.0. Delta mean fluorescence intensity (ΔMFI) was calculated by subtracting the MFI of untreated cells (mock) from the MFI of treated cells (ΔMFI = MFI_treated_ − MFI_mock_).

### 2.5. Statistical Analysis

All experiments were performed for three different replicates or with 3 independent donors. Differences in mRNA integrity, percentage eGFP positive (eGFP+) cells, and ΔMFI between experimental conditions were statistically analyzed using an ordinary one-way ANOVA with Tukey’s multiple comparison test. GraphPad Prism 10 was used for data comparison, graphical edition, and performance of statistical analyses. *p*-values below 0.05 were considered statistically significant.

## 3. Results

### 3.1. SM-LNP Outperformed ALC-LNP and MC3-LNP, Showing Results Comparable to EP

**Figure 1:**
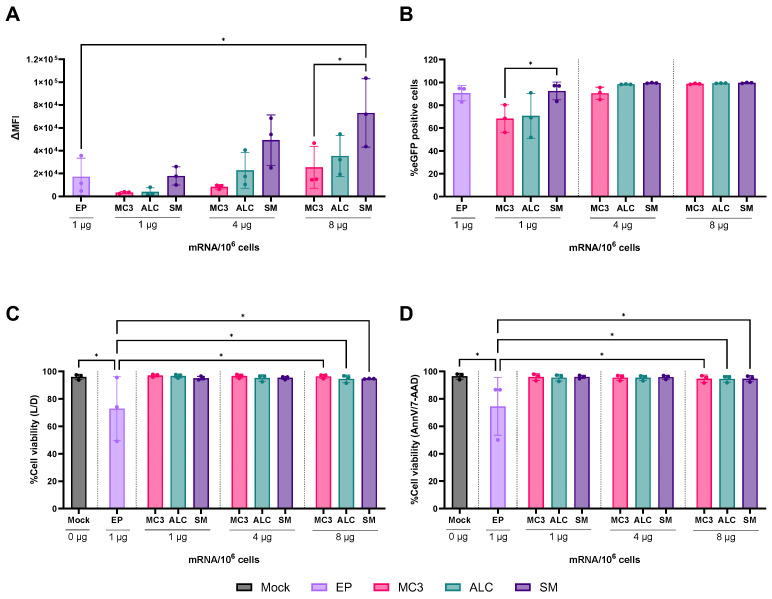
SM-LNP was comparable to EP at the same dose of eGFP mRNA with 5moU. Amount of expressed eGFP within a cell, presented as ∆MFI (**A**), and percentage of eGFP-expressing cells (**B**) appeared to be dose-dependent for all LNP formulations, although not significantly different. SM-LNP performed better out of all LNP treatments. It achieved almost 100% eGFP+ cells already at the lowest dose of 1 µg and significantly increased ∆MFI at eight times the dose of EP, while maintaining cell viability. (**C**,**D**) All LNP treatments showed comparable cell viability regardless of staining method and performed better than EP. ∆MFI = delta mean fluorescence intensity, ALC = ALC-0315, EP = electroporation, MC3 = D-Lin-MC3-DMA, SM = SM-102. Data is expressed as mean ± SD for three independent donors. Statistically significant differences were calculated using an ordinary one-way ANOVA with Tukey’s multiple comparison test. * = *p* < 0.05.

Ionizable lipids are known to be key components of LNPs, since they are essential to encapsulate nucleic acids and mediate endosomal escape, releasing the cargo to the cytosol [33]. Aiming to select the best candidate between MC3, ALC-0315, and SM-102 for following studies with different mRNA modifications, these three ionizable lipids were assessed for their impact on the transfection efficiency of hiDC. For that, eGFP mRNA containing the 5moU modification was encapsulated in LNPs formulated with DSPC, cholesterol, PEG-2000, and either MC3 (MC3-LNP), ALC-0315 (ALC-LNP), or SM-102 (SM-LNP). LNP quality parameters are specified for each LNP formulation in Table 1. LNP were incubated with hiDC in the presence of ApoE for a total of 48 h. Regarding ΔMFI, solely SM-LNPs could significantly surpass EP but needed an eight-fold higher dose (Figure 1A). At least four times the dose of MC3-LNP was needed to compare the percentage eGFP+ cells to EP, while ALC-LNP and SM-LNP achieved that at the lowest tested dose (1 µg) (Figure 1B). Cell viability using the LIVE/DEAD^TM^ Fixable Near-IR staining showed no significant difference between treatments. Interestingly, the viability of untreated cells or cells treated with LNP formulations was above 95%, against 74% for EP (Figure 1C). Viability variation was also higher for EP. The same trend was observed when staining with the AnnexinV/7-AAD (Figure 1D).

### 3.2. SM-LNP Containing eGFP mRNA with N1-Methylpseudouridine Surpasses ∆MFI of EP

**Figure 2:**
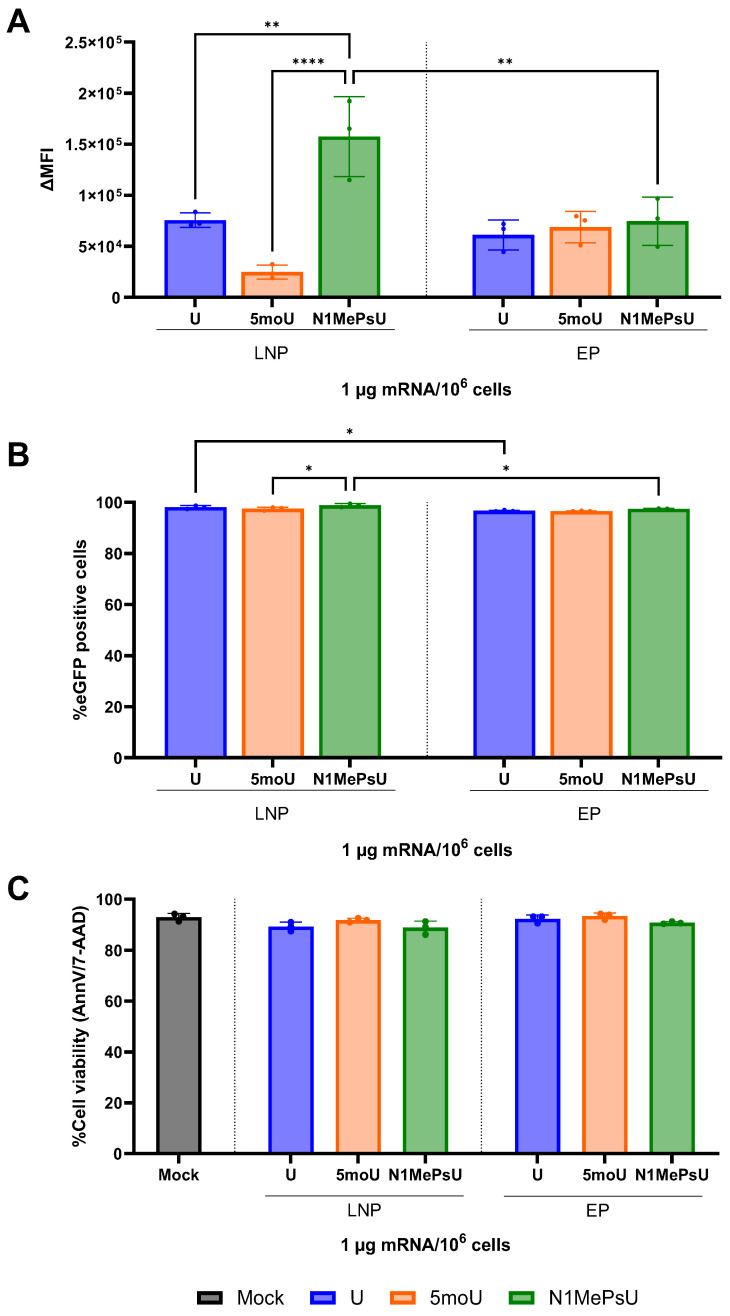
SM-LNP-mediated delivery of eGFP mRNA with N1MePsU led to the highest ∆MFI out of LNP-mediated delivery and surpassed EP. (**A**) Compared to LNPs containing other uridine modifications or to EP, SM-LNP containing eGFP mRNA with N1MePsU showed a significant increase in the amount of expressed eGFP within a cell, presented as ∆MFI. (**B**) The percentage of eGFP-expressing cells was higher for eGFP mRNA with U and N1MePsU when delivered via SM-LNP than via EP. (**C**) Cell viability was high and comparable for all tested conditions. ∆MFI = delta mean fluorescence intensity, 5moU = 5-methoxyuridine, EP = electroporation, LNP = lipid nanoparticle, N1MePsU = N1-methylpseudouridine, U = non-modified uridine. Data is expressed as mean ± SD for three independent donors. Statistically significant differences were calculated using an ordinary one-way ANOVA with Tukey’s multiple comparison test. * = *p* < 0.05, ** = *p* < 0.01 and **** = *p* < 0.0001.

After establishing the outperformance of SM-102, we selected this lipid to investigate the impact of using different uridine modifications of the eGFP mRNA sequence, since a difference in transfection efficiency related to the changes in this nucleoside has been reported for murine DC [22]. To assess if the same is achieved for human DC, eGFP mRNA containing three different uridines (U, 5moU, and N1MePsU) was encapsulated into SM-LNP and compared in vitro. Table 2 shows that LNP quality control parameters were highly similar, excluding those as confounders. Regarding ∆MFI, LNPs containing eGFP mRNA with U or 5moU performed similarly to EP. LNPs containing eGFP mRNA with N1MePsU showed significantly higher ∆MFI when compared to the other uridine modifications or to EP delivery (Figure 2A). Regardless of uridine modification, high percentages of eGFP+ cells were obtained after LNP transfection, with LNPs comparing to EP (Figure 2B). No difference between LNP and EP cytotoxicity was observed, with both treatments showing similar cell viability percentages to untreated hiDC (Figure 2C).

**Figure 3:**
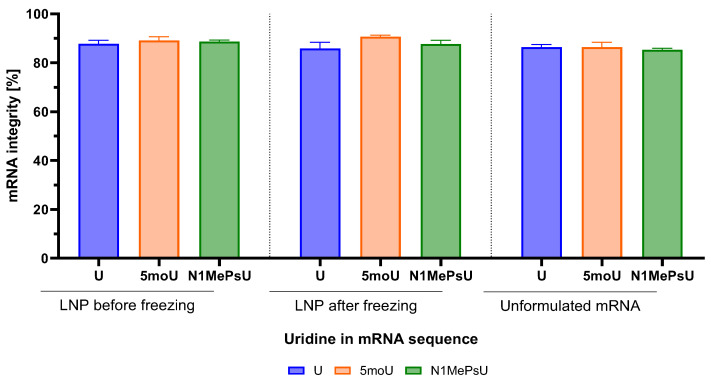
Integrity was very high for all three uridine modifications of the eGFP mRNA sequence after encapsulation in LNP. Freezing did not lead to integrity loss. 5moU = 5-methoxyuridine, LNP = lipid nanoparticle, N1MePsU = N1-methylpseudouridine, U = non-modified uridine. Data is expressed as mean ± SD for three replicates (n = 3).

To exclude mRNA integrity as the cause for differences in transfection efficiency, we performed integrity analyses for LNPs before and after freezing. Results were compared to the respective unformulated form of eGFP mRNA. Integrity of unformulated mRNA was above 80% for all uridine modifications. No decrease in mRNA integrity was observed after encapsulation, regardless of uridine modification or if LNPs were analyzed before or after freezing (Figure 3).

### 3.3. SM-LNP Containing eGFP mRNA with N1MePsU Surpassed ∆MFI of EP at the Same Dose

**Figure 4:**
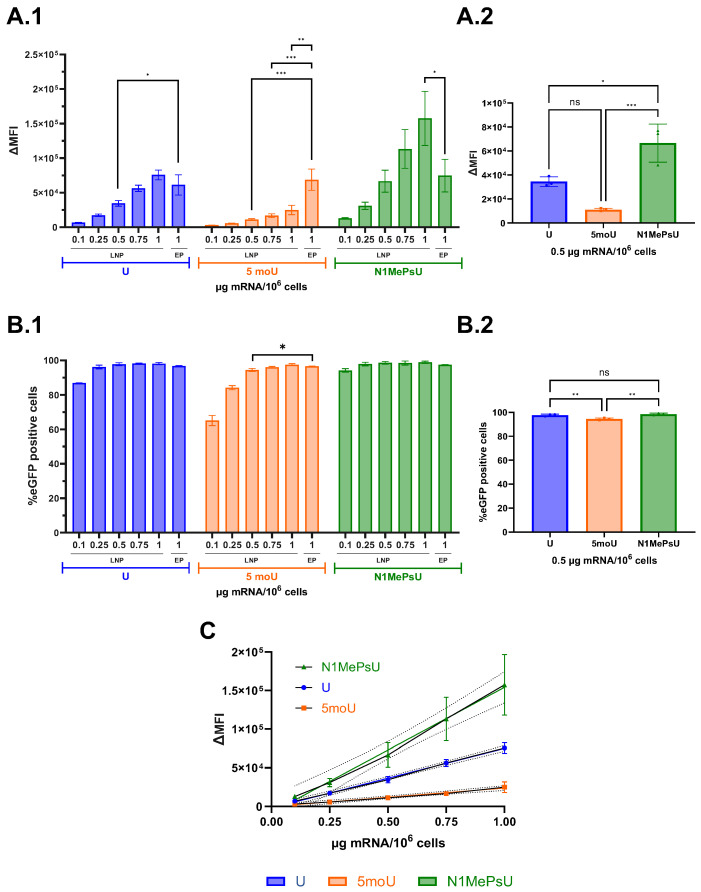
SM-LNPs containing eGFP mRNA with N1MePsU surpassed ∆MFI of EP while maintaining dose and cell viability. (**A.1**) Amount of expressed eGFP within a cell presented as ∆MFI showed SM-LNPs containing N1MePsU to significantly outperform not only EP at the same dose of 1 µg (**A.2**) but the other LNP formulations at doses from 0.5 µg on. (**B.1**) Percentage of eGFP-expressing cells of SM-LNPs containing N1MePsU was comparable to EP regardless of concentration (**B.2**) and significantly higher than SM-LNPs containing U or 5moU from 0.5 µg on. (**C**) A concentration-dependency for SM-LNP delivery of all eGFP mRNA uridine modifications was observed for ∆MFI. Dotted lines represent the confidence bands (for 95% confidence) of performed simple linear regressions. ∆MFI = delta mean fluorescence intensity, 5moU = 5-methoxyuridine, EP = electroporation, LNP = lipid nanoparticle, N1MePsU = N1-methylpseudouridine, U = non-modified uridine. Data is expressed as mean ± SD for three independent donors. Statistically significant differences were calculated using an ordinary one-way ANOVA with Tukey’s multiple comparison test. ns = non-significant, * = *p* < 0.05, ** = *p* < 0.01 and *** = *p* < 0.001.

After the promising results of combining SM-LNPs and N1MePsU eGFP mRNA, which surpassed the ∆MFI of EP at 1 µg mRNA, we saw the potential for dose sparing with this formulation. Therefore, we used the same LNP batches, to avoid variation, and compared the performance of 1 µg to the lower doses of 0.10, 0.25, 0.50, 0.75 µg mRNA/10^6^ cells. LNPs were also compared against EP at 1 µg of mRNA/10^6^ cells. Compared to EP, SM-LNPs containing eGFP mRNA with N1MePsU was the only LNP-mediated delivery to achieve a significantly higher ∆MFI at the same dose (Figure 4(A.1)). Confirming the results of Figure 2A, these LNPs outperformed SM-LNP containing either U or 5moU, showing significantly higher ∆MFI down to 0.5 µg (Figure 4(A.2)). Further, their results of percentage eGFP+ cells were comparable to EP even at the two lowest concentrations (Figure 4(B.1)). At 0.75 µg, although SM-LNP with eGFP mRNA containing 5moU showed very high percentage eGFP+ cells (94%), it was still significantly lower compared to the other two formulations (Figure 4(B.2)). A concentration-dependent effect on ∆MFI was observed for all LNP-mediated deliveries (Figure 4C). Once again, SM-LNP and EP showed similar cell viability, comparable to untreated hiDC (Figure S3).

## 4. Discussion

A typical LNP formulation is composed of four lipid components besides the cargo of interest. Each lipid serves different purposes and can influence LNP characteristics and performance [34]. Two recent reviews described how a variety of LNP formulations has been investigated for the transfection of immune cells [26,27], important for starting the immune response generation after administration of mRNA vaccines [35]. The importance of enhancing LNP transfection efficiency to DC in vitro has been demonstrated in a study using the murine DC line JAWS II, in which targeted delivery to DC via mannosylated LNPs led to a following cancer vaccine model using only one-fifth of the conventional LNPs [25]. A crucial formulation component that can impact transfection efficiency of LNPs is the ionizable lipid, important for nucleic acid encapsulation and later endosomal escape. A study investigating the influence of three ionizable lipids found in marketed LNP formulations, MC3, ALC-0315, and SM-102 were compared for mRNA transfection and protein expression efficiencies in an in vitro setup using primary murine DC and the DC2.4 mouse DC line [28].

In our work, these same lipids were investigated, aiming to increase the transfection efficiency of human DC. Despite differences in transfection, since our study was performed in the presence of ApoE and with 50 times more cells per well, our findings suggested an overall similarity to the mentioned study.

For the delivery of eGFP mRNA with 5moU, all LNP formulations showed very high cell viability (around 90%), regardless of ionizable lipid. Cell viability was confirmed with two different staining methods and suggested that the used treatments minimally induced early apoptotic cells. Since Live/Dead NIR staining relies on membrane rupture to detect non-viable cells, early apoptotic cells (AnnexinV^+^/7-AAD^−^) with intact membranes would be misclassified as viable, potentially leading to an underestimation of cell death. If early apoptosis were more prevalent after treatment, cell viability measurements would have differed between both staining methods [36], which was not the case. Initial EP cell viability (around 70%) was significantly different from LNPs and untreated cells. Interestingly, this higher cytotoxicity was not confirmed in following investigations. The reason for the difference in EP cell viability between experiments is unknown and could not be correlated with the used experimental setups or practical execution.

Regarding transfection, SM-LNP showed similar %eGFP+ cells and ∆MFI to EP at the same dose, which was not observed for MC3-LNP and ALC-LNP. Between LNP formulations, %eGFP+ cells and ∆MFI of SM-LNP were higher than those of MC3-LNP and ALC-LNP. Considering that MC3-LNP is a formulation originally designed for uptake by hepatocytes [37], it is reasonable that it underperforms in transfecting immune cells when compared to SM-102, specifically designed for an immunization vaccine. For ∆MFI, SM-LNP differed significantly from MC3-LNP and ALC-LNP. To better understand the reasons behind this apparent in vitro overperformance of SM-LNP, key mechanistic investigations would be necessary to determine how the change of ionizable lipid, the influence of pKa, and ApoE addition impact the cellular uptake by hiDC, the endosomal escape and translational processing. While these investigations would surely be interesting and help fill in many gaps on LNP mechanisms at a cellular level, they were beyond the scope of our project and cannot be seen as a guarantee of successful in vitro–in vivo translation, since in vitro studies are not capable of mimicking the dynamic forces taking place in vivo [38].

Another important component of LNPs is the cargo to be encapsulated. It has been reported that incorporation of base modifications alone might not suffice for an improvement in mRNA translation [39]. In this study, four different ionizable lipids (C12-200, cKK-E12, 2000i10 and ZA3-Ep10) were investigated for their impact on protein translation of five modified firefly luciferase-encoding mRNAs in the liver, spleen, and lung. One of the key takeaway messages of the study is the importance of carefully choosing the delivery vehicle and the nucleoside modification to achieve enhanced protein expression. Also, N1MePsU was reported as the most effective uridine modification regarding the increase in expression of firefly luciferase-encoding mRNA. Although our investigation was performed under different conditions, our results confirmed the N1MePsU sequence to have the best performance, even outperforming EP with twice the ∆MFI at the same dose. In terms of performance ranking, our investigation in human DC showed a trend of highest to lowest ∆MFI for LNP delivery of eGFP mRNA as N1MePsU > U > 5moU. Results of mRNA integrity analyses confirmed that these reported differences were not due to mRNA degradation. This same trend has already been reported for murine DC [22], although no significance was observed in both studies for the visual difference between ∆MFI of U and 5moU. Interestingly, we observed a highly efficient transfection already at an encapsulated mRNA concentration 40 times lower (94% at 0.05 µg/mL against 60% at 2 µg/mL), or an encapsulated mRNA dose per 10^6^ cells 80 times lower (0.1 µg against 8 µg). For a fair comparison, it is important to state that some setup differences exist between the investigations. While both studies used the same ionizable lipid (SM-102) and %molar of each four lipid components, we used DSPC whereas they used hydrogenated soy L-α-phosphatidylcholine. These differences in formulation seemed to lead their SM-LNP to smaller particle size, higher PDI and lower EE than ours. Also, we used a 2.5-fold higher ApoE concentration and twice the incubation time.

The comparison of different uridine modifications with SM-LNP showed no difference in cell viability between encapsulated mRNA concentrations (all around 90%) and suggested a dose-dependency of the ∆MFI. The combination of the best ionizable lipid and uridine modification allowed for high transfection efficiencies (above 90%) at doses as low as 0.1 µg/10^6^ cells. This dose is 50 times lower than the lowest dose necessary to achieve around 50% Sing15 HA positive cells with the N1MePsU modified version of influenza mRNA in another hiDC study, in which the highest %Sing15 HA positive cells achieved with SM-102 LNPs was around 60% at a dose of 15 µg/10^6^ cells [30]. A possible reason for the difference in necessary mRNA dose is the use of ApoE, which was not used in their study and has been demonstrated to improve transfection efficiency of hiDC [31] and other immune cells [16].

This study provides insights into the performance of different formulations in hiDC transfection and highlights the role of carefully combining ionizable lipid and uridine modification to achieve high in vitro transfection efficiencies with reduced LNP-delivered mRNA doses. We believe that these findings add to current understanding of overcoming the challenges associated with transfecting these immune cells and suggest strategies for reducing the in vivo dose of LNP-mRNA vaccines in future investigations.

## 5. Conclusions

To the best of our knowledge, this study reports for the first time the importance of formulation tuning for in vitro dose sparing, which can lead to future dose reduction of LNP-mRNA vaccines. Here, highly efficient transfection of human monocyte-derived dendritic cells with LNPs was achieved by combining the ionizable lipid SM-102 and eGFP mRNA containing N1-methylpseudouridine. The best LNP formulation surpassed electroporation as the gold standard for DC transfection while maintaining high cell viability. It was more effective at a lower concentration compared to previous in vitro assays reported for murine and human dendritic cells. The qualitative ranking of SM-102 over ALC-0315 and MC3 could be reproduced for hiDC, while questions remain to the correlation of transfection efficiency with an immunogenic response and the translation of in vitro and in vivo models to clinics.

## Figures and Tables

**Table 1:** Quality parameters for LNP formulations containing different ionizable lipids.

IL	HD [nm]	PDI	ζ-Potential [mV]	EE [%]	TnaR [%]	ET_c_ [EU/µg_mRNA_]
ALC-0315	89	0.05	−5.06	98	65	0.006
MC3	117	0.05	−1.58	97	87	0.004
SM-102	110	0.05	2.77	98	69	<0.004

IL = ionizable lipid, HD = hydrodynamic diameter, PDI = polydispersity index, EE = encapsulation efficiency, TnaR = total nucleic acid recovery, ET_c_ = endotoxin concentration.

**Table 2:** Quality parameters for LNP formulations containing different uridine modifications.

Uridine	HD [nm]	PDI	ζ-Potential [mV]	EE [%]	TnaR [%]	ET_c_ [EU/µg_mRNA_]
U	102	0.05	1.28	98	76	<0.003
5moU	105	0.06	1.14	97	92	0.002
N1MePsU	103	0.06	0.896	98	77	<0.005

HD = hydrodynamic diameter, PDI = polydispersity index, EE = encapsulation efficiency, TnaR = total nucleic acid recovery, ET_c_ = endotoxin concentration.

## Data Availability

The original contributions presented in this study are included in the article/Supplementary Materials. Further inquiries can be directed to the corresponding author.

## Supplementary Materials

The following supporting information can be downloaded at: https://www.mdpi.com/article/10.3390/pharmaceutics18040403/s1, Figure S1: Example for gating strategy used in flow cytometry analyses using AnnV/7AAD staining; Figure S2: Example for gating strategy used in flow cytometry analyses using L/D NIR staining and Figure S3: Cell viability was comparable between all SM-LNP formulations and EP.

## Abbreviations

The following abbreviations are used in this manuscript:

∆MFI	Delta mean fluorescence intensity
5moU	5-methoxyuridine
ALC-LNP	LNP formulated with ALC-0315
ApoE	Apolipoprotein E4
DC	Dendritic cells
EE	Encapsulation efficiency
ET_c_	Endotoxin concentration
eGFP+	eGFP positive
EP	Electroporation
fNA	Free nucleic acid
hiDC	Human monocyte-derived immature DC
IL	Ionizable lipid
LNP	Lipid nanoparticle
MC3	D-Lin-MC3-DMA
MC3-LNP	LNP formulated with MC3
mRNA	Messenger ribonucleic acid
N1MePsU	N1-methylpseudouridine
siRNA	Small interfering RNA
SM-LNP	LNP formulated with SM-102
theorNA	Theoretical nucleic acid concentration
tNA	Total nucleic acid
TnaR	Total nucleic acid recovery
U	Uridine
