# Supplementary material for: Increasing Transfection of Human Monocyte-Derived Dendritic Cells by Optimizing Lipid Nanoparticle Ionizable Lipid and mRNA Uridine Modification
Source: Pharmaceutics. 2026 Mar 25;18(4):403. doi: 10.3390/pharmaceutics18040403 (PMC13119134; doi:10.3390/pharmaceutics18040403)
Supplement: Supplementary file 1 [file pharmaceutics-18-00403-s001.zip › pharmaceutics-4192510-supplementary.pdf]

Supplementary information

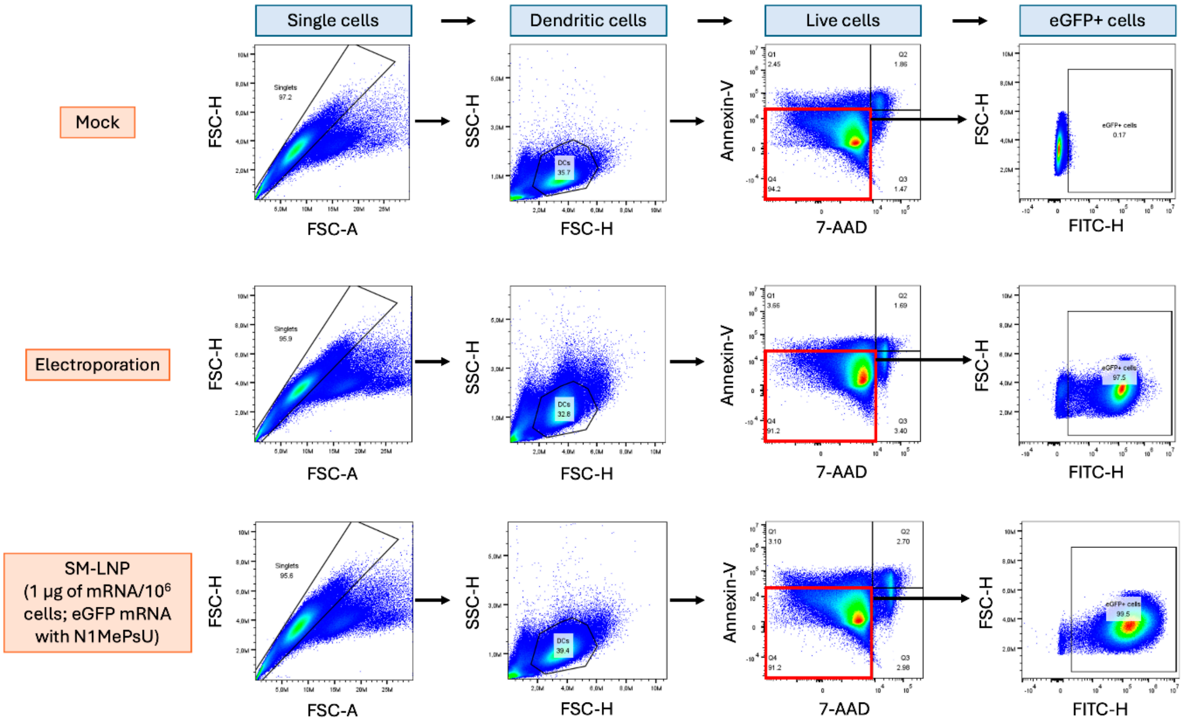

Figure S1. Example for gating strategy used in flow cytometry analyses using AnnV/7AAD staining. *Cells were first gated on forward scatter-height (FSC-H) vs forward scatter-area (FSC-A) to exclude cell aggregates (doublets) and define the single cell population. Next, cells were gated on size (FSC-H) and singularity (SCC-H) to identify dendritic cells for further analysis and exclude cell debris. The resulting subpopulation was analysed on the Annexin V-APC vs 7-AAD plot to identify the fraction viable cells (AnnV^{-}/7-AAD^{-}) within the sample analysed. Finally, live cells were gated for the expression of eGFP (FSC-H vs FITC-H). In the figure, the gating strategy is shown for three different conditions (Mock, Electroporation and LNP) of one donor only but is representative for the others.*

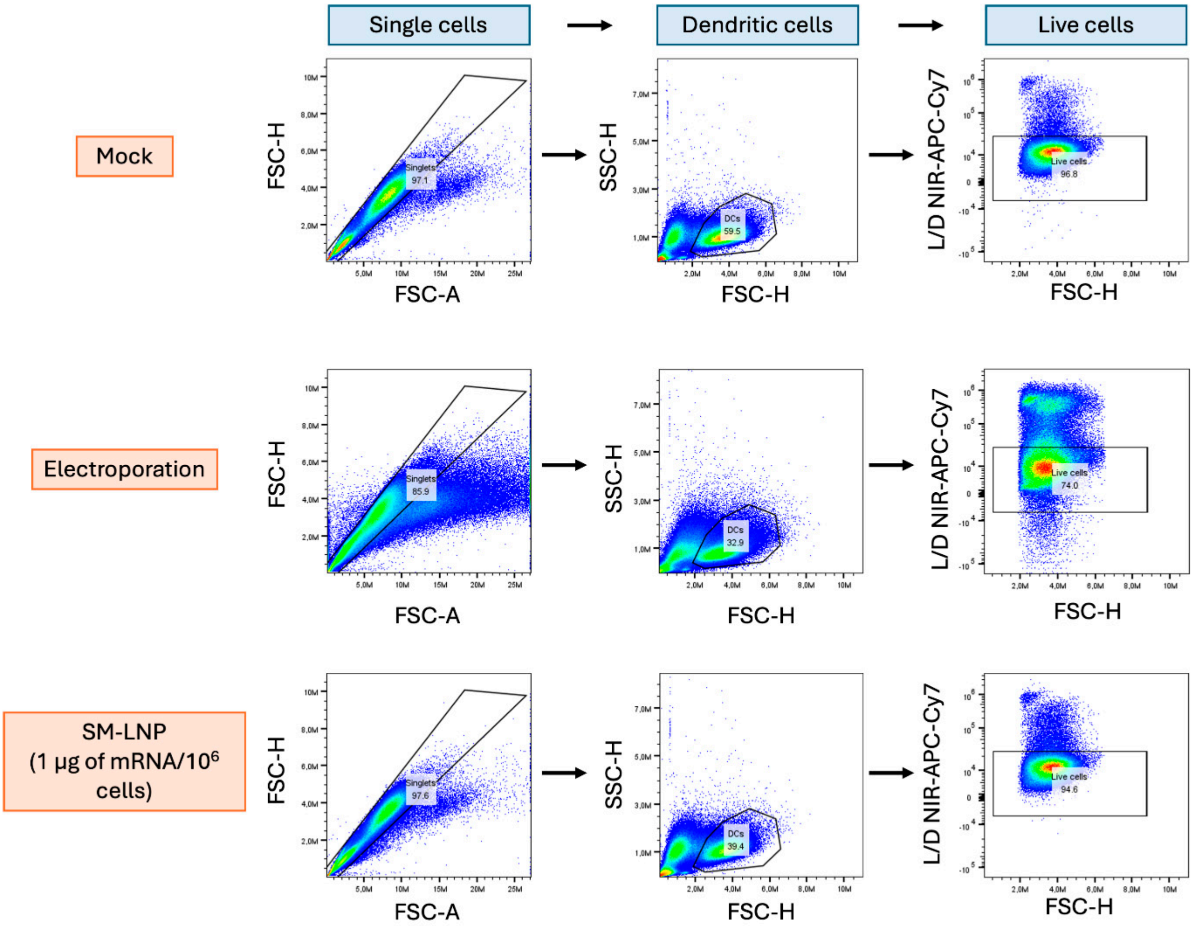

Figure S2. Example for gating strategy used in flow cytometry analyses using L/D NIR staining. *Cells were first gated on forward scatter-height (FSC-H) vs forward scatter-area (FSC-A) to exclude cell aggregates (doublets) and define the single cell population. Next, cells were gated on size (FSC-H) and singularity (SCC-H) to identify dendritic cells for further analysis and exclude cell debris. LIVE/DEAD™ Fixable Near-IR (L/D NIR) was used as the live/dead discriminator to gate APC-Cy7-negative cells, which represents the fraction of viable cells within the sample analysed. In the figure, the gating strategy is shown for three different conditions (Mock, Electroporation and LNP) of one donor only but is representative for the others.*

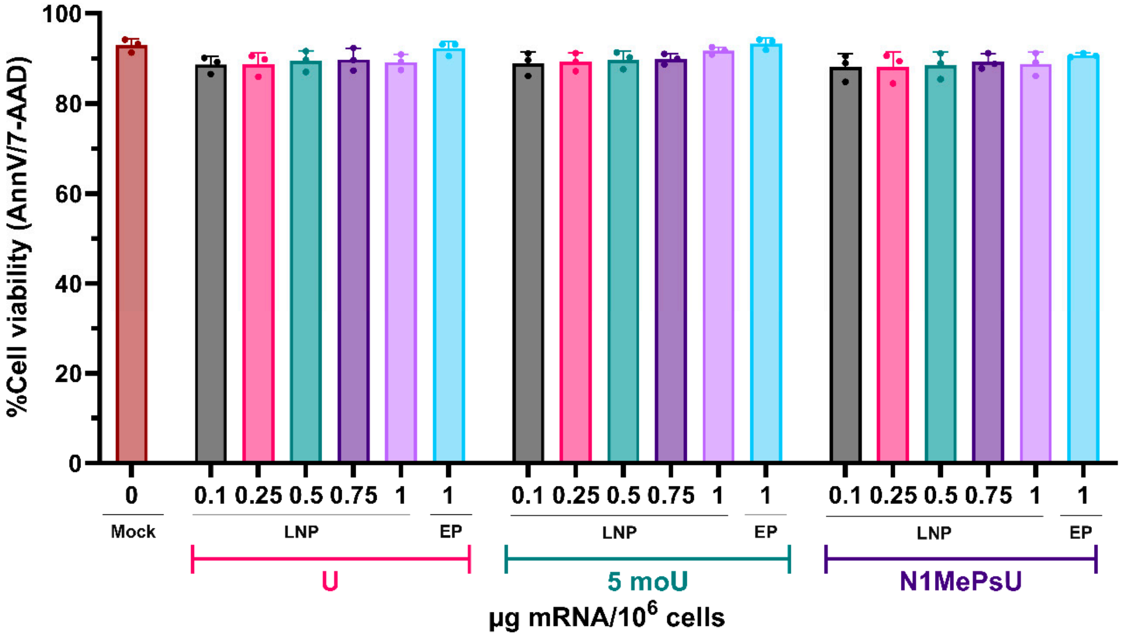

Figure S3. Cell viability was comparable between all SM-LNP formulations and EP. *Cell viability was high for all tested conditions. 5moU = 5-methoxyuridine, EP = electroporation, LNP = lipid nanoparticle, N1MePsU = N1-methylpseudouridine, U = non-modified uridine. Data are expressed as mean ± SD for three independent donors. Statistically significant differences were calculated using an ordinary one-way ANOVA with Tukey's multiple comparison test.*
